# Design, Synthesis and Biological Evaluation of 1,4-Disubstituted-3,4-dihydroisoquinoline Compounds as New Tubulin Polymerization Inhibitors

**DOI:** 10.3390/ijms160510173

**Published:** 2015-05-05

**Authors:** Ling Zhang, Yunlong Song, Jingjing Huang, Jia Liu, Wenwen Zhu, Youjun Zhou, Jiaguo Lv, Canhui Zheng, Ju Zhu

**Affiliations:** School of Pharmacy, Second Military Medical University, 325 Guohe Road, Shanghai 200433, China; E-Mails: zhanglingdd7120@163.com (L.Z.); ylsong@smmu.edu.cn (Y.S.); cpuhuangjj@126.com (J.H.); nudt_liujia@hotmail.com (J.L.); natureholic@163.com (W.Z.); zhouyoujun@smmu.edu.cn (Y.Z.); ljg19580808@163.com (J.L.)

**Keywords:** 1,4-disubstituted-3,4-dihydroisoquinoline, tubulin polymerization inhibitor, antitumor agent

## Abstract

A series of 1,4-disubstituted-3,4-dihydroisoquinoline derivatives designed as tubulin polymerization inhibitors were synthesized. Their cytotoxic activities against the CEM leukemia cell line were evaluated. Most of them displayed moderate cytotoxic activities, and compounds **21** and **32** showed good activities with *IC*_50_ of 4.10 and 0.64 μM, respectively. The most potent compound **32** was further confirmed to be able to inhibit tubulin polymerization, and its hypothetical binding mode with tubulin was obtained by molecular docking.

## 1. Introduction

**Figure 1:**
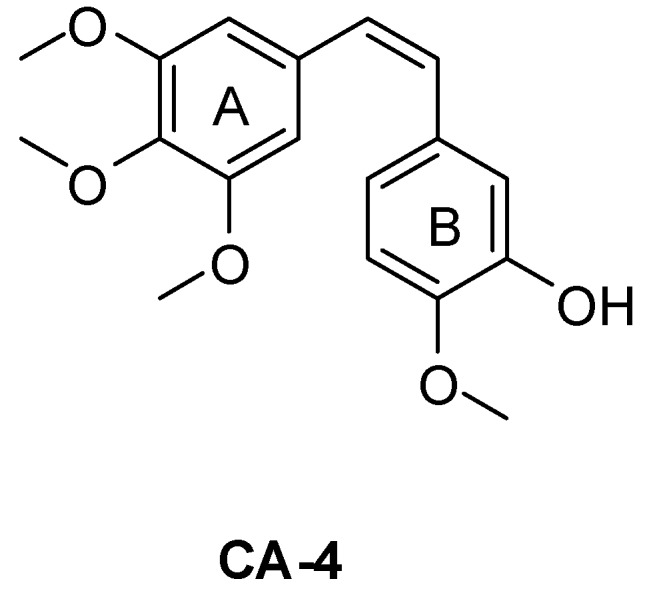
Structure of **CA-4**.

Microtubules, which are composed of the α/β heterodimeric tubulin proteins [1], have an important role in a variety of cellular process including mitosis and cell division [2,3]. The compounds, which can regulate the polymerization dynamics of the tubulin, may prove useful in the development of anticancer drugs [4,5]. They can be divided into the tubulin polymerization inhibitors such as colchicine, Combretastatin A-4 (**CA-4**) (Figure 1) and the vinca alkaloids, and microtubule stabilizing agents such as paclitaxel and epothilone [6].

**Figure 2:**
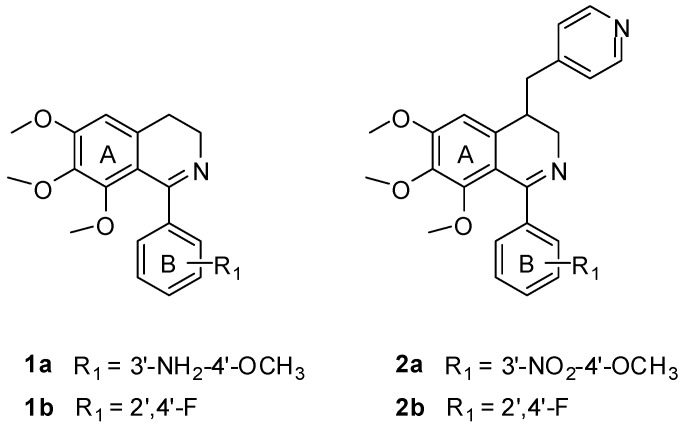
Structure of compounds **1a**, **1b**, **2a** and **2b**.

1-Phenyl-3,4-dihydroisoquinoline derivatives (e.g., compounds **1a** and **1b**) (Figure 2) designed as **CA-4** like tubulin polymerization inhibitors were synthesized by our group previously, which showed moderate cytotoxic activities [7,8]. Their docking poses in the tubulin binding site were similar to colchicine. The two phenyl rings of these inhibitors interacted with the hydrophobic P1 and P2 pocket, and another region P3 in binding site would be the potential binding site for further modification. Based on this idea, a pyridinylmethyl sidechain was introduced at the C-4 position of the isoquinoline ring which might form interactions with the region proposed by our group. As an initial attempt, some 1,4-disubstituted-3,4-dihydroisoquinoline derivatives (e.g., compounds **2a** and **2b**, Figure 2) were synthesized and found to show moderate cytotoxic activities [8]. However, no data on their inhibition of tubulin polymerization were reported. In this paper, we aim at further elucidating the structure-activity relationship (SAR) of these compounds by the syntheses and evaluation of a series of new 1,4-disubstituted-3,4-dihydroisoquinoline derivatives against human CEM leukemia cell line. The best compound was further validated for its tubulin polymerization inhibitory activity for the first time. Molecular docking studies were also performed to explain the SAR.

## 2. Results and Discussion

### 2.1. Chemistry

**Scheme 1:**
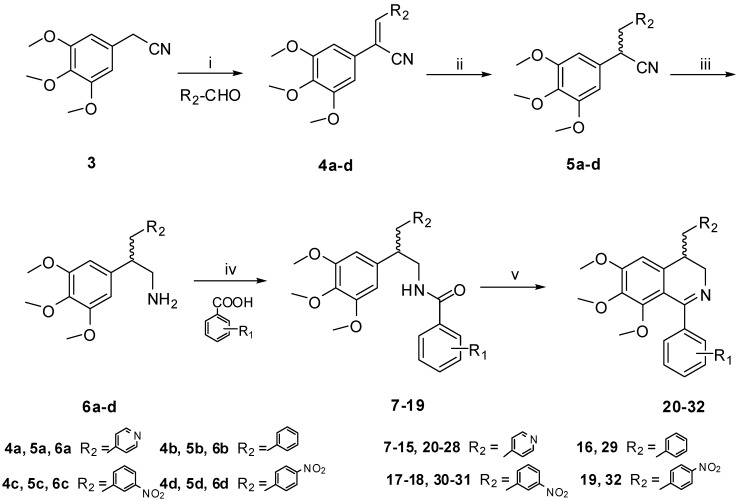
Synthetic route of the target compounds. Reagents and conditions: (i) NaOH, EtOH, 0 °C, 0.5 h, 85%; (ii) NaBH_4_, MeOH, 0 °C, 0.5 h, 86%; (iii) BF_3_, NaBH_4_, THF, 0 °C, 96%; (iv) EDC, DMAP, CH_2_Cl_2_, 25 °C, 59%–85%; (v) CH_3_CN, POCl_3_, reflux, 3 h, 63%–95%.

Thirteen new derivatives of 1,4-disubstituted-3,4-dihydroisoquinoline were synthesized. The general method used for the synthesis of the 3,4-dihydroisoquinoline derivatives was outlined in Scheme 1. The condensation of **3** with aromatic aldehyde gave **4a–d**, which were subsequently treated with NaBH_4_ to afford amines **6a–d** in good yield via independent two-step reduction reaction [9,10], then **6a–d** was reacted with the substituted benzoic acids to give the substituted benzamides **7**–**19**. The target compounds **20**–**32** were finally obtained by Bischler-Napieralski cyclization of the corresponding benzamides **7**–**19**. The chemical structures of all the target compounds (Table 1) were confirmed by ^1^H NMR and MS (ESI) data.

**Table 1:** Cytotoxic activities of the target compounds.

Compound	R_1_	R_2_	*IC*_50_ (μM)
			CEM
**20**	3',4'-OCH_3_	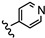	36.29
**21**	3'-NH_2_-4'-OCH_3_	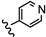	4.10
**22**	3'-NHCOCH_3_-4'-OCH_3_	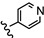	24.32
**23**	4'-OCH_3_	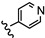	17.76
**24**	4'-OH	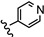	40.33
**25**	4'-OCOCH_3_	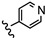	>100
**26**	4'-F	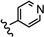	44.58
**27**	4'-CH_3_	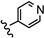	>100
**28**	3'-CH_3_	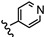	44.07
**29**	3'-NH_2_-4'-OCH_3_	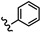	46.11
**30**	4'-OH	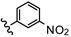	32.48
**31**	2',4'-F	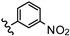	3.08
**32**	2',4'-F	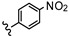	0.64
**2a**	3'-NO_2_-4'-OCH_3_	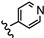	29.25
**2b**	2',4'-F	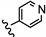	15.21
**Colchicine**	/	/	0.004

### 2.2. In Vitro Cytotoxic Activity

The cytotoxicities of the target compounds were evaluated against human CEM leukemia cell line by the MTT assay (Table 1). The cytotoxic activity of **21** (*IC*_50_ = 4.10 μM) was slightly higher than that of **1a** (*IC*_50_ = 6.92 μM), which has the same substitutions in the B ring. This indicated that introducing a pyridin-4-ylmethyl moiety to the C-4 position of the isoquinoline ring of the lead compounds was in favor of activity. On the contrary, introducing a benzyl moiety to this position was detrimental to the activity, which was demonstrated by the reduced activity of compound **29** than **1a**. However, further introduction of a nitro group to the 4-benzyl can significantly improve the cytotoxic activity. Compounds **31** (*IC*_50_ = 3.08 μM) and **32** (*IC*_50_ = 0.64 μM) which bear 3'-nitro and 4'-nitro at the benzyl group are 13-fold and 61-fold more potent than **1b** (*IC*_50_ = 39.15 μM), which has the same substitution in the B ring. The results also confirmed that 4'-nitro had better activities than 3'-nitro. Compound **32** showed more than 20-fold improvement than previously reported structurally related compound **2b** (*IC*_50_ = 15.21 μM) in which there was only a pyridinylmethyl sidechain at the C-4 position. Based on these results, it showed that introducing a polar sidechain to the C-4 position of the isoquinoline ring of the lead compounds was a practical strategy to increase their cytotoxic activities.

The substituents of the B ring are also very important to the activity. This was evidenced by compound **23**, which has a 4'-OCH_3_ in the B ring. It showed more active than **24**, **25**, **26** and **27** which have 4'-OH, 4'-OCOCH_3_, 4'-F and 4'-CH_3_ substituted B-rings. An amino group at the 3'-position of the B-ring is necessary for high cytotoxic activity. The potent compound **21** bears 3'-NH_2_ group, while 3'-OCH_3_ and 3'-NHCOCH_3_ substituted compounds, such as **20** and **22**, showed significantly lower activities. Compared with the reported compound **2a** (*IC*_50_ = 29.25 μM), the cytotoxic activity of compound **21** was about 7-fold more potent against CEM leukemia cell line. Generally, the electron-donating groups in the B ring were more favorable to the cytotoxic activities, which is consistent with the SARs of the **CA-4** derivatives [11].

### 2.3. In Vitro Tubulin Polymerization Inhibitory Activity

**Figure 3:**
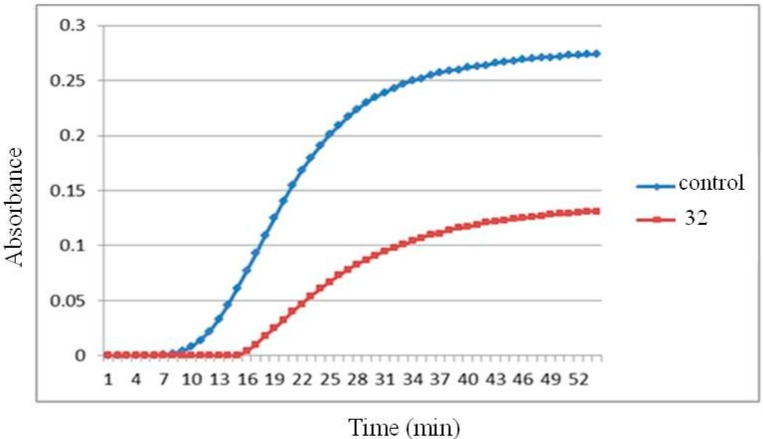
Tubulin polymerization inhibition of compound **32** at 40 µM.

To test whether these 1,4-disubstituted-3,4-dihydroisoquinoline compounds could inhibit tubulin polymerization, the most potent compound **32** was subjected to evaluation by the tubulin assembly assay. Compound **32** showed inhibitory effect (52%) of microtubule assembly from pure tubulin at 40 μM level (Figure 3).

### 2.4. Molecular Docking Study

**Figure 4:**
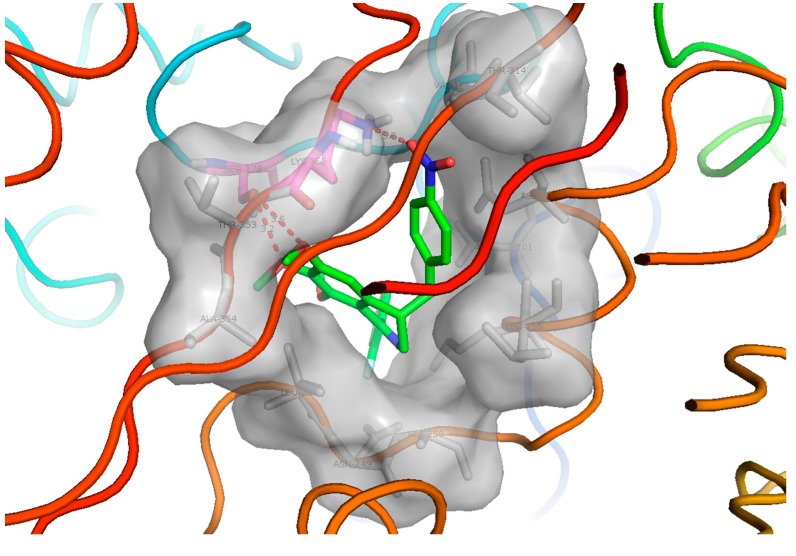
The hypothetical binding mode of the *S* isomer of compound **32** to tubulin protein. P1 and P2 are the two hydrophobic pockets, and P3 is a polar region in the interface between α/β-tubulin. The figure was generated using PyMol (http://pymol.souceforge.net/).

To further elucidate the binding mode with tubulin, molecular docking studies were performed on the most potent compound **32**. The X-ray crystal structure of the DAMA-colchicine-tubulin complex (PDB entry: 1SA0) was used as the tubulin protein template. All the modeling studies were performed by using Discovery Studio 3.5 molecular simulation package. It is noted that the synthesized compounds were racemates. Therefore, the two isomers of compound **32** were both docked into the target. From the docking results, the *S* isomer of the compound **32** clearly had better intereactions with the target than the *R* isomer. The predictive docking mode of the *S* isomer of **32** to tubulin protein was found to be similar to that of colchicines, as shown in Figure 4. In its docking mode, the two phenyl rings of compound **32** interacted with the hydrophobic P1 and P2 pocket, which were the most important two pharmacophores of the tubulin polymerization inhibitors. The 4'-nitro benzyl moiety interacted with the region P3 as expected. The 6,7-di-methoxy group formed two hydrogen bonds with Ser178 of tubulin. It is also interesting to see an oxygen atom of the 4'-nitro group formed a hydrogen bond with the amino group at the flexible sidechain of Lys352, which exactly explain why the introduction of 4'-nitro benzyl to the C-4 position of the isoquinoline ring of the lead compounds is favorable to activity.

## 3. Experimental Section

### 3.1. Chemistry

The melting point was determined on a XT4A microscope melting-point apparatus (Keyi Electron Optical Instrument Factory, Beijing, China) without correction.^1^H NMR and ^13^C NMR spectra were recorded on BRUKER AVANCE 300 and 600 spectrometers (Bruker Company, Rheinstetten, Germany), with TMS as an internal standard and CDCl_3_ as the solvent. ESI mass spectra were performed on an API-3000 LC-MS spectrometer (Applied Biosystems, Toronto, ON, Canada). Flash column chromatography was performed with silica gel 300–400 mesh (Qingdao Haiyang Chemical, Qingdao, China). All solvents and reagents were purchased from commercial suppliers and, when necessary, were purified and dried by standard protocols. Organic solutions were dried over anhydrous sodium sulfate. The purity of the final compounds was assessed with an Agilent 1200 HPLC (Agilent Technologies, Santa Clara, CA, USA), and the results were greater than 95%.

**(*Z*)-3-(Pyridin-4-yl)-2-(3,4,5-trimethoxyphenyl)acrylonitrile (4a)**

A mixture of 2-(3,4,5-trimethoxyphenyl)acetonitrile **3** (0.52 g, 2.5 mmol) and isonicotinaldehyde (0.54 g, 5.0 mmol) was dissolved in EtOH (10 mL) at 0 °C, and then NaOH (0.10 g, 2.5 mmol) was added to the solution mixture. After stirring for 30 min, the solution was filtrated and the residue was collected under suction filtration and dried to afford compound **4a** (0.63 g, 85.04%) as a yellow green crystals. mp 134–135 °C. ^1^H NMR (300 MHz, CDCl_3_): δ 3.91 (s, 3H), 3.43 (s, 3H), 3.95 (s, 6H), 6.90 (s, 2H), 7.40 (s, 1H), 7.71 (dd, 2H, *J* = 1.5, 4.8 Hz), 8.75 (dd, 2H, *J* = 1.5, 4.5 Hz).

The synthetic methods for the intermediates **4b**–**d** were similar to the synthesis of intermadiate **4a**.

**3-(Pyridin-4-yl)-2-(3,4,5-trimethoxyphenyl)propanenitrile (5a)**

A mixture of **4a** (0.62 g, 2.1 mmol), NaBH_4_ (0.32 g, 8.5 mmol) and 20 mL MeOH was heated under 50 °C for 0.5 h. The mixture was evaporated and the residue diluted with 25 mL EtOAc. The organic layer was dried and filtered and the solvent removed by evaporation. After the solution was cooled and stayed overnight to give the compound **5a** (2.18 g, 86.21%) as a white crystals. mp 129–130 °C. ^1^H NMR (300 MHz, CDCl_3_): δ 3.10–3.24 (m, 2H), 3.82 (s, 6H), 3.85 (s, 3H), 3.99 (t, 1H), 6.40 (s, 2H), 7.09 (d, 2H, *J* = 4.5 Hz), 8.56 (d, 2H, *J* = 4.5 Hz).

The synthetic methods for the intermediates **5b**–**d** were similar to the synthesis of intermadiate **5a**.

**3-(Pyridin-4-yl)-2-(3,4,5-trimethoxyphenyl)propan-1-amine (6a)**

BF_3_·O(C_2_H_5_)_2_ (7.5 mmol) was slowly added to a stirred solution of **5a** (0.76 g, 2.5 mmol) and NaBH_4_ (10 mmol) in THF (10 mL) at 0 °C. The solution was refluxed for 1 h, poured into water, and extracted with EtOAc (15 mL × 3). The combined extracts were dried over anhydrous Na_2_SO_4_ and filtered. The solvents were removed by evaporation to afford **6a** (0.72 g, 96.61%) as a yellow oil. ^1^H NMR (300 MHz, CDCl_3_): δ 2.96–2.79 (m, 5H), 3.81 (s, 6H), 3.83 (s, 3H), 6.32 (s, 2H), 6.98 (dd, 2H, *J* = 1.5, 4.5 Hz), 8.43 (dd, 2H, *J* = 1.5, 4.5 Hz).

The synthetic methods for the intermediates **6b**–**d** were similar to the synthesis of intermadiate **6a**.

**3,4-Dimethoxy-*N*-(3-(pyridin-4-yl)-2-(3,4,5-trimethoxyphenyl)propyl)benzamide (7)**

A mixture of 3,4-dimethoxybenzoic acid (2.1 mmol), EDC·HCl (0.54 g, 2.8 mmol), DMAP (0.03 g, 0.28 mmol) and CH_2_Cl_2_ (20 mL), was stirred at room temperature for 10 min, Then **6a** (0.73 g, 2.4 mmol) dissolved in CH_2_Cl_2_ (10 mL) was added through a dropping funnel for about 5 min. After stirring for about 30 min, the mixture was washed by water (30 mL × 2) and saturated aqueous Na_2_CO_3_ (30 mL × 2). The organic layer was dried over anhydrous MgSO_4_ and filtered. Then the filtrate was concentrated by evaporation and 7 (0.59 g, 52.68%) was obtained as white powder. ^1^H NMR (300 MHz, CDCl_3_): δ 2.92–3.04 (m, 2H), 3.08–3.21 (m, 1H), 3.38–3.44 (m, 1H), 3.78 (s, 6H), 3.83 (s, 3H), 3.90 (s, 6H), 3.95–4.02 (m, 1H), 5.93–6.01 (m, 1H), 6.35 (s, 2H), 6.81–6.98 (m, 2H), 7.01–7.05 (m, 2H), 7.31 (s, 1H), 8.45 (dd, 2H, *J* = 1.5, 4.2 Hz).

The synthetic methods for the intermediates **8**–**19** were similar to the synthesis of intermadiate **7**.

**1-(3,4-Dimethoxyphenyl)-6,7,8-trimethoxy-4-(pyridin-4-ylmethyl)-3,4-dihydroisoquinoline (20)**

A mixture of 7 (0.70 g, 1.5 mmol), POCl_3_ (0.82 mL, 9 mmol) and CH_3_CN (15 mL) was stirred and heated under reflux for 4 h, then the solvents were removed by evaporation, and the residue was dissolved in EtOAc (30 mL). Then the solution was neutralied to pH = 7 with saturated aqueous Na_2_CO_3_ and washed by water (30 mL × 3). The organic layer was dried over anhydrous MgSO_4_ and filtered. Then the filtrate was concentrated under reduced pressure, The residue after evaporation was purified by flash chromatography on silica gel (eluent: CH_2_Cl_2_/MeOH = 100:1 *v*/*v*) and crystallized from EtOAc to give **20** (0.13 g, 19.3%) as white powder. mp 121–122 °C. ^1^H NMR (300 MHz, CDCl_3_): δ 2.77–2.80 (m, 3H), 3.43 (s, 3H), 3.47 (dd, 1H, *J* = 4.8, 14.7 Hz), 3.76 (s, 3H), 3.80 (s, 3H), 3.93 (s, 3H), 3.94 (s, 3H), 4.04 (dd, 1H, *J* = 3.0, 14.4 Hz), 6.20 (s, 1H), 6.88 (d, 1H, *J* = 8.4 Hz), 7.02 (dd, 1H, *J* = 1.8, 5.1 Hz), 7.07 (dd, 2H, *J* = 1.5, 4.5 Hz), 7.17 (d, 1H, *J* = 1.8 Hz), 8.51 (dd, 2H, *J* = 1.5, 4.5 Hz). ESI-MS (*m*/*z*): 449.3 [M + 1]^+^.

The synthetic methods for the compounds **21**–**32** were similar to the synthesis of compound **20**.

**2-Methoxy-5-(6,7,8-trimethoxy-4-(pyridin-4-ylmethyl)-3,4-dihydroisoquinolin-1-yl)aniline (21)**

Yellow crystal. mp 109–110 °C. ^1^H NMR (300 MHz, CDCl_3_): δ 2.74–2.84 (m, 3H), 3.40–3.46 (m, 4H), 3.75 (s, 3H), 3.79 (s, 3H), 3.82 (s, 2H, N–H), 3.89 (s, 3H), 4.03 (dd, 1H, *J* = 2.4, 14.7 Hz), 6.18 (s, 1H), 6.79 (d, 1H, *J* = 8.1 Hz), 6.86 (dd, 1H, *J* = 1.8, 8.1 Hz), 6.97 (d, 1H, *J* = 1.8 Hz), 7.06 (dd, 2H, *J* = 1.5, 4.5 Hz), 8.50 (dd, 2H, *J* = 1.5, 4.5 Hz). ESI-MS (*m*/*z*): 434.3 [M + 1]^+^.

***N*-(2-Methoxy-5-(6,7,8-trimethoxy-4-(pyridin-4-ylmethyl)-3,4-dihydroisoquinolin-1-yl)phenyl)acetamide (22)**

White powder, mp 173–176 °C. ^1^H NMR (300 MHz, CDCl_3_): δ 2.18 (s, 3H), 2.75–2.86 (m, 3H), 3.44 (s, 3H), 3.45 (dd, 1H, *J* = 4.2, 15.0 Hz), 3.76 (s, 3H), 3.80 (s, 3H), 3.94 (s, 3H), 4.04 (dd, 1H, *J* = 2.4, 15.0 Hz), 6.20 (s, 1H), 6.93 (d, 1H, *J* = 8.4 Hz), 7.08 (dd, 2H, *J* = 1.2, 4.5 Hz), 7.32 (d, 1H, *J* = 9.0 Hz), 7.77 (s, 1H), 8.48 (s, 1H), 8.49 (dd, 2H, *J* = 1.5, 4.5 Hz). ESI-MS (*m*/*z*): 476.3 [M + 1]^+^.

**6,7,8-Trimethoxy-1-(4-methoxyphenyl)-4-(pyridin-4-ylmethyl)-3,4-dihydroisoquinoline (23)**

White powder, mp 118–119 °C. ^1^H NMR (300 MHz, CDCl_3_): δ 2.75–2.87 (m, 3H), 3.40 (s, 3H), 3.47 (dd, 1H, *J* = 4.5, 14.7 Hz), 3.75 (s, 3H), 3.80 (s, 3H), 3.86 (s, 3H), 4.05 (dd, 1H, *J* = 2.7, 15.0 Hz), 6.19 (s, 1H), 6.90–6.95 (m, 2H), 7.06 (dd, 2H, *J* = 1.5, 4.2 Hz), 7.45–7.50 (m, 2H), 8.50 (dd, 2H, *J* = 1.5, 4.2 Hz). ESI-MS (*m*/*z*): 419.3 [M + 1]^+^.

**4-(6,7,8-Trimethoxy-4-(pyridin-4-ylmethyl)-3,4-dihydroisoquinolin-1-yl)phenol (24)**

White crystal, mp 140–141 °C. ^1^H NMR (300 MHz, CDCl_3_): δ 3.75–3.90 (m, 3H), 3.38 (s, 3H), 3.36 (dd, 1H, *J* = 4.8, 15.0 Hz), 3.77 (s, 6H), 4.01 (dd, 1H, *J* = 2.4, 14.7 Hz), 6.24 (s, 1H), 6.65 (d, 2H, *J* = 8.7 Hz), 7.06 (dd, 2H, *J* = 1.2, 4.5 Hz), 7.30 (d, 2H, *J* = 8.4 Hz), 8.46 (dd, 2H, *J* = 1.2, 4.5 Hz). ESI-MS (*m*/*z*): 405.2 [M + 1]^+^.

**4-(6,7,8-Trimethoxy-4-(pyridin-4-ylmethyl)-3,4-dihydroisoquinolin-1-yl)phenyl acetate (25)**

White powder, mp 119–120 °C. ^1^H NMR (300 MHz, CDCl_3_): δ 2.32 (s,3H), 2.75–2.89 (m, 3H), 3.40 (s, 3H), 3.48 (dd, 1H, *J* = 4.5, 15.0 Hz), 3.76 (s, 3H), 3.79 (s, 3H), 4.07 (dd, 1H, *J* = 2.7, 15.0 Hz), 6.21 (s, 1H), 7.07 (dd, 2H, *J* = 1.5, 4.5 Hz), 7.11–7.16 (m, 2H), 7.49–7.53 (m, 2H), 8.51 (dd, 2H, *J* = 1.8, 4.5 Hz). ESI-MS (*m*/*z*): 447.2 [M + 1]^+^.

**1-(4-Fluorophenyl)-6,7,8-trimethoxy-4-(pyridin-4-ylmethyl)-3,4-dihydroisoquinoline (26)**

White powder, mp 91–92 °C. ^1^H NMR (300 MHz, CDCl_3_): δ 2.75–2.90 (m, 3H), 3.39 (s, 3H), 3.48 (dd, 1H, *J* = 4.8, 15.0 Hz), 3.76 (s, 3H), 3.80 (s, 3H), 4.06 (dd, 1H, *J* = 3.0, 15.0 Hz), 6.20 (s, 1H), 7.05–7.12 (m, 4H), 7.46–7.53 (m, 2H), 8.51 (dd, 2H, *J* = 1.5, 4.5 Hz). ESI-MS (*m*/*z*): 407.2 [M + 1]^+^.

**6,7,8-Trimethoxy-4-(pyridin-4-ylmethyl)-1-*p*-tolyl-3,4-dihydroisoquinoline (27)**

Yellow crystal, mp 142–143 °C. ^1^H NMR (300 MHz, CDCl_3_): δ 2.40 (s, 3H), 2.74–2.89 (m, 3H), 3.38 (s, 3H), 3.47 (dd, 1H, *J* = 4.5, 14.7 Hz), 3.75 (s, 3H), 3.79 (s, 3H), 4.05 (dd, 1H, *J* = 2.7, 15.0 Hz), 6.19 (s, 1H), 7.06 (dd, 2H, *J* = 1.5, 4.5 Hz), 7.19–7.22 (m, 2H), 7.39–7.42 (m, 2H), 8.50 (dd, 2H, *J* = 1.5, 4.5 Hz). ESI-MS (*m*/*z*): 403.2 [M + 1]^+^.

**6,7,8-Trimethoxy-4-(pyridin-4-ylmethyl)-1-*m*-tolyl-3,4-dihydroisoquinoline (28)**

White powder, mp 138–139 °C. ^1^H NMR (300 MHz, CDCl_3_): δ 2.40 (s, 3H), 2.76–2.86 (m, 3H), 3.39 (s, 3H), 3.47 (dd, 1H, *J* = 4.5, 15.0 Hz), 3.76 (s, 3H), 3.78 (s, 3H), 4.07 (dd, 1H, *J* = 2.4, 14.7 Hz), 6.19 (s, 1H), 7.07 (dd, 2H, *J* = 1.5, 4.5 Hz), 7.19–7.31 (m, 3H), 7.33 (s, 1H), 8.50 (dd, 2H, *J* = 1.5, 4.5 Hz). ESI-MS (*m*/*z*): 403.3 [M + 1]^+^.

**5-(4-Benzyl-6,7,8-trimethoxy-3,4-dihydroisoquinolin-1-yl)-2-methoxyaniline (29)**

White powder, mp 96–97 °C. ^1^H NMR (300 MHz, CDCl_3_): δ 2.73–2.84 (m, 3H,), 3.44 (dd, 1H, *J* = 4.5, 14.4 Hz), 3.45 (s, 3H), 3.73 (s, 3H), 3.76–3.85 (m, 2H, N–H), 3.79 (s, 3H), 3.89 (s, 3H), 4.03 (dd, 1H, *J* = 2.4, 14.7 Hz), 6.18 (s, 1H), 6.79 (d, 1H, *J* = 8.4 Hz), 6.87 (dd, 1H, *J* = 2.1, 8.1 Hz), 6.99 (d, 1H, *J* = 2.1 Hz), 7.10–7.15 (m, 2H), 7.17–7.22 (m, 1H), 7.24–7.30 (m, 2H). ESI-MS (*m*/*z*): 433.3 [M + 1]^+^.

**4-(6,7,8-Trimethoxy-4-(3-nitrobenzyl)-3,4-dihydroisoquinolin-1-yl)phenol (30)**

Yellow powder, mp 201–203 °C. ^1^H NMR (300 MHz, CDCl_3_): δ 2.84–2.91 (m, 3H), 3.38 (s, 3H), 3.42–3.46 (m, 1H), 3.78 (s, 3H), 3.81 (s, 3H), 3.99 (d, 1H, *J* = 14.5 Hz), 6.32 (s, 1H), 6.60 (d, 2H, *J* = 8.5 Hz), 7.31 (d, 2H, *J* = 8.5 Hz), 7.36–7.41 (m, 2H), 8.05–8.06 (m, 2H). IR (KBr, cm^−1^) *ν*: 2940.81, 2839.79, 1593.73, 1523.77, 1351.70, 1317.11, 1247.45, 1127.89, 837.23. ^13^C NMR (500 MHz, CDCl_3_): δ 37.36, 39.66, 49.27, 56.07, 60.95, 61.47, 106.05, 115.18, 121.42, 123.78, 128.69, 129.28, 131.91, 136.03, 139.03, 141.47, 141.61, 148.34, 152.98, 155.99, 158.36, 166.59. ESI-MS (*m*/*z*): 449.2 [M + 1]^+^.

**1-(2,4-Difluorophenyl)-6,7,8-trimethoxy-4-(3-nitrobenzyl)-3,4-dihydroisoquinoline (31)**

White powder, mp 112–113 °C. ^1^H NMR (300 MHz, CDCl_3_): δ 2.85–2.96 (m, 3H), 3.43 (s, 3H), 3.52 (dd, 1H, *J* = 4.2, 15.0 Hz), 3.73 (s, 3H), 3.78 (s, 3H), 4.14 (d, 1H, *J* = 14.4 Hz), 6.14 (s, 1H), 6.80–6.87 (m, 1H), 6.94–7.00 (m, 1H), 7.35–7.45 (m, 2H), 7.53 (d, 1H, *J* = 7.2 Hz), 8.02–8.10 (m, 2H). ESI-MS (*m*/*z*): 469.2 [M + 1]^+^.

**1-(2,4-Difluorophenyl)-6,7,8-trimethoxy-4-(4-nitrobenzyl)-3,4-dihydroisoquinoline (32)**

Yellow crystal, mp 215–216 °C. ^1^H NMR (300 MHz, CDCl_3_): δ 2.85–2.96 (m, 3H), 3.43 (s, 3H), 3.52 (dd, 1H, *J* = 4.2, 15.3 Hz), 3.71 (s, 3H), 3.78 (s, 3H), 4.09–4.16 (m, 1H), 6.10 (s, 1H), 6.80–6.87 (m, 1H), 6.94–7.00 (m, 1H), 7.24–7.27 (m, 2H), 7.53 (dd, 1H, *J* = 8.1, 14.7 Hz), 8.14 (d, 2H, *J* = 8.7 Hz). ESI-MS (*m*/*z*): 469.2 [M + 1]^+^.

### 3.2. In Vitro Cytotoxic Activity

Cell culture. Cytotoxic effects were examined in the CEM human leukemia cell lines. Cancer cells were all cryopreservated and passaged by Pharmacological Laboratory of Shanghai Institute of pharmaceutical industry. The culture medium was DMEM with 10% fetal bovine serum (FBS) and double antibody. Culture was maintained at 37 °C with 5% CO_2_ in a humidified atmosphere.

*In vitro* cytototoxic assay. The cytotoxicity was determined using MTT dye reduction assay. All tested compounds were dissolved in DMSO (Merck, Darmstadt, Germany) and was diluted with PBS. For each well of a 96-well microplate, 100 μL of cell dilution was seeded, allowed to attach overnight, and then exposed to varying concentrations (10^−4^–10^−9^ M) of compounds for 72 h maintained at 37 °C in 5% CO_2_. The plated was incubated for 4h after 20 μL of MTT (5 mg/mL) was added to each well. The resultant tetrazolium salt was dissolved in 100 μL dimethylsulfoxide and read at 570 nm using a microplate reader (Varioskan Flash; Thermo Fisher Scientific, Waltham, MA, USA).

Compounds were tested in triplicate in at least three independent assays. The *IC*_50_ values were determined from the linear portion of the curves by a nonlinear regression analysis. Average values were reported.

### 3.3. In Vitro Tubulin Polymerization Inhibitory Activity

Bovine brain tubulin was purchased from Cytoskeleton Inc. (Denver, CO, USA). Tubulin (>99% pure, 2 mg/mL) in 100 μL of general tubulin buffer (80 mM Na-Pipes, pH 6.9, 1 mM EGTA, 1 mM MgCl_2_, 1 mM GTP, and 5% glycerol) at 0 °C was placed in a pre-warmed 96-well plates at 37 °C in the presence of tested compounds at varying concentrations. The reaction was started by warming the samples at 37 °C. The mass of polymer formed was monitored by turbidimetry at 340 nm every 1 min for 45 min with a BioTek’s Synergy 4 multifunction microplate reader (Winooski, VT, USA) [12,13,14].

## 4. Conclusions

In summary, thirteen 1,4-disubstituted-3,4-dihydroisoquinoline derivatives were synthesized and evaluated for their anti-proliferative activities against human CEM leukemia cell line with the aim of elucidating the SARs of the lead compounds discovered earlier. Most of them displayed moderate cytotoxic activities, and compound **21** and **32** showed good activities. The SARs of the synthesized compounds were investigated. It was found that the electron-donating substituents in the B ring were more favorable to the cytotoxic activities. The most potent compound **32** was confirmed to be able to inhibit tubulin polymerization and its hypothetical binding mode to tubulin was proposed by molecular docking. Some compounds had more potent cytotoxic activities than the structurally related compounds reported in our paper. Further optimization of these compounds is ongoing in our laboratory.
